# A Tailored Thermosensitive PLGA-PEG-PLGA/Emulsomes Composite for Enhanced Oxcarbazepine Brain Delivery via the Nasal Route

**DOI:** 10.3390/pharmaceutics10040217

**Published:** 2018-11-05

**Authors:** Ghada M. El-Zaafarany, Mahmoud E. Soliman, Samar Mansour, Marco Cespi, Giovanni Filippo Palmieri, Lisbeth Illum, Luca Casettari, Gehanne A. S. Awad

**Affiliations:** 1 Department of Pharmaceutics and Industrial Pharmacy, Faculty of Pharmacy, Ain Shams University, Monazzamet Elwehda Elafrikeya Street, Abbaseyya, Cairo 11566, Egypt; ghada@pharma.asu.edu.eg (G.M.E.-Z.); mahmoud.e.soliman@pharma.asu.edu.eg (M.E.S.); samar.mansour@guc.edu.eg (S.M.); gawad@pharma.asu.edu.eg (G.A.S.A.); 2 Department of Pharmaceutical Technology, Faculty of Pharmacy and Biotechnology, German University in Cairo, Al-Tagmoaa Alkhames, Cairo 11835, Egypt; 3 School of Pharmacy, University of Camerino, Via S. Agostino 1, 62032 Camerino (MC), Italy; marco.cespi@unicam.it (M.C.); gianfilippo.palmieri@unicam.it (G.F.P.); 4 IDentity, 19 Cavendish Crescent North, The Park, Nottingham NG7 1BA, UK; lisbeth.illum@illumdavis.com; 5 Department of Biomolecular Sciences, School of Pharmacy, University of Urbino, Piazza del Rinascimento 6, 61029 Urbino (PU), Italy

**Keywords:** nose to brain delivery, antiepileptic drug, drug delivery, block copolymers, thermogel system

## Abstract

The use of nanocarrier delivery systems for direct nose to brain drug delivery shows promise for achieving increased brain drug levels as compared to simple solution systems. An example of such nanocarriers is emulsomes formed from lipid cores surrounded and stabilised by a corona of phospholipids (PC) and a coating of Tween 80, which combines the properties of both liposomes and emulsions. Oxcarbazepine (OX), an antiepileptic drug, was entrapped in emulsomes and then localized in a poly(lactic acid-*co*-glycolic acid)-poly(ethylene glycol)-poly(lactic acid-*co*-glycolic acid) (PLGA-PEG-PLGA) triblock copolymer thermogel. The incorporation of OX emulsomes in thermogels retarded drug release and increased its residence time (MRT) in rats. The OX-emulsome and the OX-emulsome-thermogel formulations showed in vitro sustained drug release of 81.1 and 53.5%, respectively, over a period of 24 h. The pharmacokinetic studies in rats showed transport of OX to the systemic circulation after nasal administration with a higher uptake in the brain tissue in case of OX-emulsomes and highest MRT for OX-emulsomal-thermogels as compared to the IN OX-emulsomes, OX-solution and Trileptal^®^ suspension. Histopathological examination of nasal tissues showed a mild vascular congestion and moderate inflammatory changes around congested vessels compared to saline control, but lower toxic effect than that reported in case of the drug solution.

## 1. Introduction

Hydrogels that are prepared from “intelligent” or “smart” polymers and capable of exhibiting a physicochemical response in a nonlinear manner to an external stimuli, such as temperature [1,2], pH [3], light [4], counter-ion changes [5] and biological molecules [6] have been extensively investigated in the literature. These polymers have great potential in drug delivery and tissue engineering [7,8,9], in addition to sensors and valves [10]. 

Thermosensitive polymers are a subcategory of smart polymers that undergo temperature-induced reversible sol-gel transition upon heating/cooling of the aqueous polymer solution. Drugs/formulations sequestered within these copolymers by simple mixing are administered as a solution that gels at body temperature at the desired site of application [11], and are used particularly in the field of controlled drug release.

Thermosensitive di-block copolymers that are composed of hydrophilic polyethylene glycol (PEG) as block A and hydrophobic biodegradable polyesters as block B e.g., poly(lactic acid) (PLA); poly(glycolic acid) (PGA); polyethylene terephthalate (PET); and polycaprolactone (PCL), have been studied as controlled release drug carriers [12,13,14]. Particularly, the di-block copolymer PEG/PLGA hydrogels are attractive polymers for the delivery of drugs since they are biodegradable with a good safety profile and their compositions can be tailored to provide sustained drug delivery [15,16,17]. 

Tri-block thermosensitive copolymers are used in either ABA or BAB configuration, with the poloxamers (ABA) (PEG-PPO-PEG) being of interest due to their commercial availability in a wide range of molecular weights and block ratios [18]. However, they show slow biodegradation kinetics and the inability to provide sustained drug delivery over more than just a few days [19,20]. Therefore, triblock copolymers consisting of PEG and PLGA copolymer moieties are regarded as good alternatives, being non-toxic, water-soluble biocompatible, biodegradable according to copolymer ratio used, and thus, can have tailor-made properties [21].

**Figure 1:**
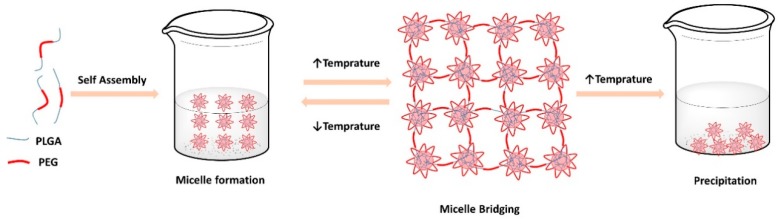
Schematic diagram of the sol-gel transition of BAB type poly(lactic acid-*co*-glycolic acid)-poly(ethylene glycol)-poly(lactic acid-*co*-glycolic acid) (PLGA-PEG-PLGA) tri-block copolymer aqueous solution in response to temperature.

The gelation mechanisms of thermosensitive hydrogels based on PLGA-PEG-PLGA are illustrated in Figure 1. The cited copolymer self-assembles in micellar formations in aqueous media. A rise in temperature results in the dehydration of the PEG chains, causing the micelle formations to shrink, thus enhancing the interaction between the PEG and PLGA blocks [22]. At temperatures below the critical gelation temperature (CGT), the bridging between micelles are usually unstable due to the low hydrophobicity of PLGA [23]. With increasing temperatures that approach CGT and exceed it, a firm bridged micellar network forms because of the increase in hydrophobic interaction between the chains, thus enhancing inter micellar interaction and aggregation, leading to subsequent gelation [24,25]. 

In the present study we adopted a PLGA-PEG-PLGA tri-block copolymer (PLGA (3:1) *M*_W_ 1.88 kDa and PEG *M*_W_ 1.5 kDa) that was first synthesized by Zentner et al., 2001. Gelling of an aqueous solution of a PLGA-PEG-PLGA copolymer is initiated at around 32 °C, while at 37 °C, the flow of the copolymer solution decreases dramatically as the gel state is established, maintaining the gel in situ for prolonged periods of time. Hence, it is an excellent candidate for prolonging residence time of the sequestered drug/formulation on the nasal mucosa after IN application [26].

Oxcarbazepine (OX), which is used as a model drug in this study, is employed for the treatment of partial and generalized seizures that are associated with epilepsy. OX is usually administered via the oral route exhibiting distribution to non-targeted tissues, a high first pass metabolism, with potential drug-drug interactions as well as undesirable side effects [27]. OX is a microsomal enzyme inducer that necessitates the use of high therapeutic doses which support the interest in a direct transport of the drug to the brain such as what may be achieved by nose to brain delivery [28].

Emulsomes are nanocarriers prepared with lipid cores either in the solid or liquid crystalline state at 25 °C, coated with a phospholipid bilayer. These nanocarriers combine the characteristics of lipid spheres (apolar core) and liposomes (hydrophilic surface) [29]. Due to the apolar solid core, emulsomes can entrap higher amounts of lipophilic drugs [30]. Being composed of triglycerides that are surrounded by phospholipids, emulsomes form particles similar to micelles, hence, vastly increasing the solubility, as well as the bioavailability of poorly soluble drugs [31]. Previous studies from our laboratories optimised the emulsomes in terms of physicochemical characteristics, especially in terms of a prolonged release profile and nose to brain delivery of OX. It was found that the emulsomes delivery system showed a significantly higher brain *C*_max_ and a higher brain AUC_0–1440min_ than an IV injection of OX and the oral administration of the marketed OX product. 

The aim of this study was to investigate whether a combination of the thermo-responsive hydrogel and lipid-based nanoparticles (emulsomes) would enhance the promising results obtained previously for the OX emulsomes alone in delivery of the drug from nose to brain. 

In the first instance the thermogelling PLGA-PEG-PLGA copolymer was synthesized and characterized using NMR, GPC, DSC, DLS, and its rheological behaviour investigated. Oxcarbazepine (OX)-loaded emulsomes was than mixed with PLGA-PEG-PLGA tri-block copolymer to develop a 2-phase drug depot system and the formed thermogel was characterized in terms of sol-gel transition temperature, viscosity, drug release and mucoadhesion.

Finally, the delivery system was evaluated in rats for the transport of OX from the nasal cavity to the systemic circulation and the brain tissue, and the histopathology of the OX-emulsome-thermogel delivery system in the nasal cavity investigated.

## 2. Materials and Methods 

### 2.1. Materials

Oxcarbazepine (OX) was a gift from Novartis Pharmaceutical Co. (Cairo, Egypt). Triolein (TO) (Captex GTO) was generously supplied by Abitec Corp. (Janesville, WI, USA). Soya phosphatidylcholine (PC) and Tween 80 (Tw) were purchased from Sigma Chemical Co. (St. Louis, MO, USA). Chloroform and methanol were purchased from ADWIC, El Nasr Pharmaceutical Co. (Cairo, Egypt). The dialysis membrane made from standard grade regenerated cellulose (molecular weight cut off 12–14 Da) was purchased from Spectrum Laboratories Inc. (Rancho Dominguez, CA, USA). Di-hydroxyl-terminated polyethylene glycol (*M*_w_ 1.5 kDa) (PEG 1.5 kDa) and stannous-2-ethyl-hexanoate were purchased from Sigma-Aldrich (Steinheim, Germany). 3,6-dimethyl-1,4-dioxane-2,5-dione (d,l-lactide) and 1,4-dioxane-2,5-dione (glycolide) were kindly supplied by PURAC Biochem (Gorinchem, Netherlands). All other chemical used were of analytical grade.

### 2.2. Synthesis of PLGA-PEG-PLGA Tri-Block Copolymers

The composition and choice of molecular weights of the PEG and the PLGA components of the PLGA-PEG-PLGA copolymer was designed to have similar sol-gel forming properties as those that were previously synthesized by Chen et al. [32]. They found that the gel-to-sol and sol-to-gel transition only occurred with appropriate molecular weight and molar mass dispersity of the copolymer.

The PLGA-PEG-PLGA copolymer was produced by the method described by Zentner et al. [7]. Briefly, PEG 1.5 kDa was initially dried by azeotropic distillation in toluene under an atmosphere of nitrogen, followed by drying at 130 °C under vacuum, then allowed to melt by placing 6 g in a three-neck flask under vacuum with continuous agitation at150 °C for 3 h, thus generating a viscous liquid mass [33]. d,l-lactide and glycolide were then added with respective amounts of 6 and 1.5 g to maintain a fixed molar ratio of 3:1.

The reaction mixture was maintained at 150 °C under vacuum for another 30 min with constant mixing to allow the fusion of d,l-lactide and glycolide. Stannous-2-ethyl-hexanoate was subsequently added and the reaction mixture was further heated for 1 h [7].

In order to remove the unreacted monomers at the end of the reaction, the raw copolymer was dissolved in dichloromethane and poured dropwise into ethyl ether kept in an ice bath. The pure copolymer was precipitated, then separated by filtration, dried under vacuum and kept refrigerated [34].

### 2.3. Characterization of PLGA-PEG-PLGA Copolymers

In order to verify polymer formation and determine its chemical structure and molecular weight, the following characterisation methods were utilized on PLGA-PEG-PLGA copolymer solutions:

#### 2.3.1. Nuclear Magnetic Resonance (^1^H-NMR)

Structural analysis of the PLGA-PEG-PLGA copolymer, initial determination of its molecular weight and estimation of the lactide:glycolide ratio, was carried out via NMR spectroscopy. The copolymer was dissolved in deuterated acetone and analysis was done using NMR spectroscopy (Varian Mercury 400 MHz spectrometer, Varian Inc., Palo-Alto, CA, USA) operating at 400 MHz [35]. Subsequently, the molecular weight was calculated by comparing the integral of the signal for PEG protons chemical shift at 3.6 ppm with the integrals of glycolide at 4.8 ppm and those of lactide at 1.6 and 5.2 ppm.

#### 2.3.2. Gel Permeation Chromatography (GPC) 

7.5 mg of the PLGA-PEG-PLGA copolymer was dissolved in tetrahydrofuran and acetonitrile and then placed in a water bath at 40 °C for one hour. Vial contents were filtered through a regenerated cellulose syringe filter (diameter 13 mm and 0.45 µm pore size) then analyzed by a high performance liquid chromatography system (HPLC, Agilent Series 1100 LCsystem (Agilent Technologies, Waldbronn, Germany), equipped with a Gel permeation column (TSKGel 2500HHR from Tosoh Bioscience LLC, Montgomeryville, PA, USA) maintained at 37 °C, with tetrahydrofuran as the eluent at a flow rate of 1 mL/min [36]. 

The molecular weight was quantified by use of a calibration curve resulting from a series of PEG standards with low polydispersity and known molecular weights ranging from 106 Da to 21.3 kDa (PEG Calibration Kit PL 2070-0100, Varian Inc., Palo-Alto, CA, USA). Data were analyzed using the software DATAAPEX (DataApex Ltd., Prague, Czech Republic). Accordingly, the molecular weights of the copolymer were computed, expressed as number average molecular weight, *M*_n_, and weight average molecular weight, *M*_w_. 

#### 2.3.3. Differential Scanning Calorimetry (DSC)

Thermal analysis of the PLGA-PEG-PLGA copolymer was carried out in a DSC apparatus (PerkinElmer 8500, Perkin Elmer, Waltham, MA, USA) equipped with an intracooler in inert nitrogen atmosphere. A sample of the copolymer was placed in a closed aluminium pan and heated with a two-stage program at a rate of 10 °C/min. The 1st heating run was conducted from ambient temperature to 150 °C to remove the thermal history of the polymer (i.e., remaining solvent and humidity) and the 2nd run from −40 to 210 °C to determine the sample specific data. Both heating cycles were separated by a cooling run that was conducted at a rate of 10 °C/min [37].

#### 2.3.4. Size Measurement of Copolymer Solution by Dynamic Light Scattering (DLS)

The DLS equipment (Zetasizer Nano ZS, Malvern Instrument, Malvern, UK) was adopted to measure the hydrodynamic diameter and size distribution of the PLGA-PEG-PLGA copolymer micelles, using a detector laser beam fixed at an angle of 90° [38]. To investigate the formation of micelles and the variation in micellar size in relation to temperature, a copolymer solution of 20% *w/w* in deionized water was prepared. 1 mL of the sample was analyzed at increasing temperatures from 10 to 40 °C. The experiment was performed in triplicate.

### 2.4. Preparation and Characterization of PLGA-PEG-PLGA Thermogel Solutions Loaded with Emulsomes

#### 2.4.1. Sample Preparation

An optimised emulsome formulation (TO17-TW) was chosen from previous studies comprising a 3:1 ratio of PC:TG (TG ~ Triolein), a total lipid content of 30 mg and coated with Tween 80 [36,37]. The emulsomes were prepared and characterized as a lipid mixture of PC and TO, as previously described [39,40]. Briefly, 10 mgs OX were introduced into a clean, dry round bottom flask, dissolved in chloroform, and then mixed with 30 mg lipid mixture composed of PC:TG (3:1). The organic solvent was removed using a rotary evaporator (Model RVO5, Janke and Kunkel, IKA Laboratories, Staufen, Germany). The formed thin film was hydrated by agitation for 1 h at room temperature with phosphate buffer (pH 6.8) containing 5% tween 80. The formed dispersion was sonicated for 1 min (Bath sonicator model 275T, Crest Ultrasonics Carp, Trenton, NJ, USA), then extruded through 450 nm cellulose nitrate membranes, and stored at 4 °C. The size of the emulsomes has previously been found to be in the order of 101.5 ± 2.9 nm [40]. 

For characterization of the final thermogel, different formulation concentrations (5, 10, 20, and 30% *w*/*w*) of aqueous PLGA-PEG-PLGA copolymer solutions were investigated. The solutions were prepared by dissolving pre-cooled PLGA-PEG-PLGA copolymer in deionized water via continuous magnetic stirring of the mixture in an ice bath until complete dissolution. This was followed by the addition of emulsomes in different loading concentrations (0, 10, 25, and 50% *v/v*) by physical mixing. Table 1 shows the coding, compositions and gelation temperature of all prepared thermogel formulations with G1-G16 indicating the individual compositions.

#### 2.4.2. Macroscopic Phase Behaviour of Aqueous Copolymer Solutions and Determination of Gelation Temperature

The sol-gel transition temperatures of the aqueous copolymer solutions with the addition of 0–50% emulsomes were investigated by a tube inversion method [38]. Briefly, 0.5 mL of each solution (G1-G16) was placed in a thermoset circulating water bath where the temperature increased stepwise (0.5 °C) from 20 to 52 °C, with an equilibrium time of 3 min. At each temperature setting, tubes were inverted to check the flow properties. Solutions were considered to be in the gel state if the appearance changed from transparent to opaque with no observed flow for 30 s following the tube inversion [41].

#### 2.4.3. Viscosity Measurement

The viscosities of the copolymer solutions that completely gelled at a temperature ranging between room (25 °C) and body temperatures (37 °C) were measured using a programmable Brookfield viscometer (Brookfield engineering laboratories Inc. model HADV-II, Middleboro, MA, USA) connected to a digital thermostatically controlled water bath (Polyscience Inc. model 9101, Niles, IL, USA). A sample of 0.5 g thermogel was applied onto the lower plate of the viscometer, adjusted at 37 °C. Rheological measurements were conducted at rpm range of 1–100 (shear rate 2–200 s^−1^), using CPE-52 spindle.

### 2.5. In Vitro Drug Release

Franz type diffusion cells (Variomag Telesystem, H+P Labortechnik, Oberschleißheim, Germany) were used to compare the release and transport of OX from OX-emulsomes [40] to that from OX-emulsomal gel (G16) through a dialysis membrane in. An amount of the OX-emulsomes or the OX-emulsomal gel equivalent to 3 mg OX was introduced to the donor compartment of the diffusion cell. The detailed methodology was as described elsewhere [40]. 

### 2.6. Mucoadhesion Studies

The mucoadhesive strengths of the G16 emulsomal thermogel in comparison to its corresponding plain thermogel G4 and 0.5% Carbopol 980 gel as a positive control were determined by measuring the force that is required to detach the formulation from freshly excised sheep nasal mucosa, using a texture analyser CT3 (Brookfield Engineering Laboratories, Inc., Middleboro, MA, USA) in tension mode. The sheep nasal mucosa was obtained from the local slaughter house. The mucosal membrane was separated from underlying fat and loose tissues, then washed with distilled water followed by phosphate buffer (pH 6.8) at 37 °C and cut into pieces that fit the stainless-steel cylindrical probe of the device of 10 mm in diameter (3.14 cm^2^). 

A cut sample of the nasal mucosa was glued to the upper probe using a cyanoacrylate adhesive, keeping the mucosal side exposed. Fixed amounts of the appropriate thermogel were attached to the lower probe of the instrument with double-sided adhesive tape. The upper probe with the nasal tissue was lowered until the tissue contacted the surface of the gelled sample with a downward trigger force of 4 N for a contact time of 5 min to ensure intimate contact between the tissues and the samples [42]. The probe was then moved vertically upwards at a constant speed of 3 mm/s [43]. Work of adhesion, mJ, peak detachment force, N, deformation peak, mm, and final load, N, were all calculated from force-distance plots using the built-in Texture Exponent software.

### 2.7. In Vivo Studies

#### 2.7.1. Pharmacokinetic Study

• Administration of OX containing formulations to rats

Male Wister albino rats weighing 200–250 g were divided into two groups, each containing forty eight rats. Group 1 received OX IN in phosphate buffer solution (pH 6.8) intranasally (IN), Group 2 received IN OX-emulsomes dispersion, Group 3 received IN G16 emulsomal thermogel and Group 4 received Oral Trileptal suspension. The drug dose was adjusted to be 0.32 mg/kg.

Results obtained from Group 2 and Group 4 were taken from identical experiments reported in El-Zaafarany et al. [40] for comparative reasons.

Conscious, non-anaesthetised rats were fixed in a prostrate position and 80 µL/kg of the formulations were administered into each of the two nostrils using a microinjector [44]. Blood samples were collected from the retro-orbital vein of four rats at predetermined time intervals and introduced into heparinized tubes. Plasma was separated from blood samples by centrifugation at 4000 rpm for 10 min and OX was analysed using a LC-MS/MS technique [40]. At each time interval, the animals were sacrificed and the brain tissues (not including the olfactory bulb) were separated, homogenized with saline and stored at −80°C until further assay. Ethical approval of this protocol was given by the “Experiments and Advanced Pharmaceutical Research Unit” of the Faculty of Pharmacy, Ain Shams University, Cairo, Egypt and National Institutes of Health guidelines for the use and care of Laboratory animals were followed in the course of this study (Project No.: 6 10 2016 approved on December 2016).

• Assay of OX content in plasma and brain samples

Chromatographic conditions (LC–MS/MS analysis parameters) and pharmacokinetic analysis of OX in plasma and brain (Peak plasma and brain concentrations (*C*_max_), the time to reach these peaks (*t*_max_) were determined, area under OX concentration-time curve (AUC_0–2880min_), time to reach half the maximum plasma concentration (*t*_1/2_), and the mean residence time (MRT)), as reported in our previous publication [40].

#### 2.7.2. Histopathological Study

20 μL of the Ox loaded G16 emulsomal thermogel was applied daily to one nostril of male Wister rats (group size *n* = 3) weighing 180–220 g for 14 consecutive days, while the other nostril was used as control and treated with normal saline. Rats were then sacrificed and the nasal septa with their covering membranes were separated, formalin-fixed for one day, decalcified, washed with tap water, and then dehydrated by sequential exposure to methyl, ethyl and absolute alcohol).

Specimens were cleared by xylene, embedded in paraffin blocks, sectioned by slide microtome. Sections were deparaffinized and stained with hematoxylin and eosin and examined with a light microscope (Olympus optical microscope, Tokyo, Japan).

### 2.8. Statistical Analysis

Experiments were done in triplicates and all data that are shown in this study are expressed as a mean ± standard deviation (SD). Comparison of means was undertaken using Student *t*-test for dual comparisons and ANOVA test followed by Tukey-Kramer post-Hoc test for multiple comparisons using a statistical software program (GraphPad Instat, La Jolla, CA, USA). *p* < 0.05 were considered to be statistically significant.

## 3. Results

### 3.1. Preparation and Characterization of PLGA-PEG-PLGA Triblock Copolymers

PLGA-PEG-PLGA triblock copolymers were synthesized via ring-opening polymerization of d,l-lactide and glycolide initiated with PEG. The copolymer formed appeared as a sticky yellowish-brown semisolid. PLGA-PEG-PLGA is a non-ionic triblock copolymer consisting of hydrophilic PEG segments and hydrophobic PLGA segments. The process of polymer synthesis is shown in the supplementary part Figure S1.

#### 3.1.1. NMR Spectroscopy

A typical NMR spectrum of PLGA-PEG-PLGA triblock copolymer is shown in the supplementary part Figure S2, showing the same characteristic signals as those reported in the literature [37]. Respective methyl (–CH_3_) and methine (–CH–) proton peaks pertaining to lactide monomer were observed at 1.6 and 5.2 ppm. A peak at 4.8 ppm, corresponding to methene protons (–CH_2_–) of glycolide monomer was obtained, as well as a peak at 3.6 ppm relative to methene in PEG monomer which was used for the calculation of the PEG:PLGA ratio. 

By comparing the peak areas of specific types of prominent protons at corresponding chemical shifts or by the total number of these protons, the molecular weight of the polymer was calculated [36,37,45]. By analyzing the value of the integrals of peaks that are related to lactide and glycolide, the chain number molecular weight (*M*_n_) of the tri-block copolymer was determined to be 5.2 kDa

#### 3.1.2. GPC

Figure S3 in the supplementary part shows the GPC chromatograms of PEG and the prepared PLGA-PEG-PLGA block copolymer. It is obvious that the copolymer was eluted at a shorter time (at 6.76 min) than PEG, indicating that polymerization was successful and products of lower retention time have been formed. In addition, the unimodal GPC curve reflects that the purity of the prepared copolymer is sufficiently high. The number average molecular weight (*M*_n_) was found to be 4.4 kDa, which corresponds to that obtained by NMR. Also, the weight average molecular weight (*M*_w_) was determined to be 7.6 kDa, with a calculated PDI of 1.69.

#### 3.1.3. DSC

DSC thermograms shown in Figure S4 of the supplementary part illustrate that PEG had a melting point at 49.2 °C with a melting enthalpy of 160.5 J/g. On the other hand, PLGA-PEG-PLGA exhibited glass transition at a temperature of −13.3 °C, which is comparable to what has been reported in the literature of PEG containing copolymers that show crystallization in the range of −25 to 0 °C [46]. 

#### 3.1.4. DLS

Copolymers composed of hydrophilic and hydrophobic portions can when in solution be present both as individual hydrated molecules or as spherical aggregates (micelles) [37]. When these copolymers are exposed to aqueous conditions, the hydrophilic PEG and glycol portions will be oriented on the outside of the micelle in contact with water, and the hydrophobic PLGA parts will be located in the inner part of the micelle due to their hydrophobic intermolecular interactions. Thus, self-assembled micelles are formed [47]. Hence, DLS can be used as a method to highlight the transition of non-hydrated molecules to micelles i.e., copolymer association.

Figure S5 of the supplementary part shows the average micellar sizes of PLGA-PEG-PLGA copolymer samples that were prepared at 20% *w/v* and measured by DLS at temperatures ranging from 10–40 °C. No significant differences were encountered in the micellar sizes of the copolymer samples measured in the tested temperature range, as indicated by nearly overlapping peaks, always achieving results comparable to those relating to micellar systems (in the range of 4–20 nm). The observed larger size of samples measured at temperatures between 35–40 °C may be regarded as indication of system aggregation due to thermogelation [48,49]. 

### 3.2. Preparation and Characterization of Plain and Emulsome-Loaded PLGA-PEG-PLGA Thermogel Solutions

In order to study the physicochemical characteristics of PLGA-PEG-PLGA triblock copolymer solutions, either plain or emulsome-loaded, the solutions were prepared in different concentrations of the copolymer (5, 10, 20, and 30% *w/w*). Likewise, emulsomes were loaded into the copolymer solutions in concentrations of 0, 10, 25, and 50% *v/v*. For all of the prepared thermogel solutions, sol-gel transition temperature and viscosity were measured. Rheological properties of plain thermogels were studied as shown in the supplementary part (Figure S6).

#### 3.2.1. Thermogelation of the Copolymer Solutions

The phase transition temperatures of thermogel solutions G1 to G16 were evaluated using the inverted tube method by increasing temperatures from 20 to 52 °C while checking phase changes. 

**Figure 2:**
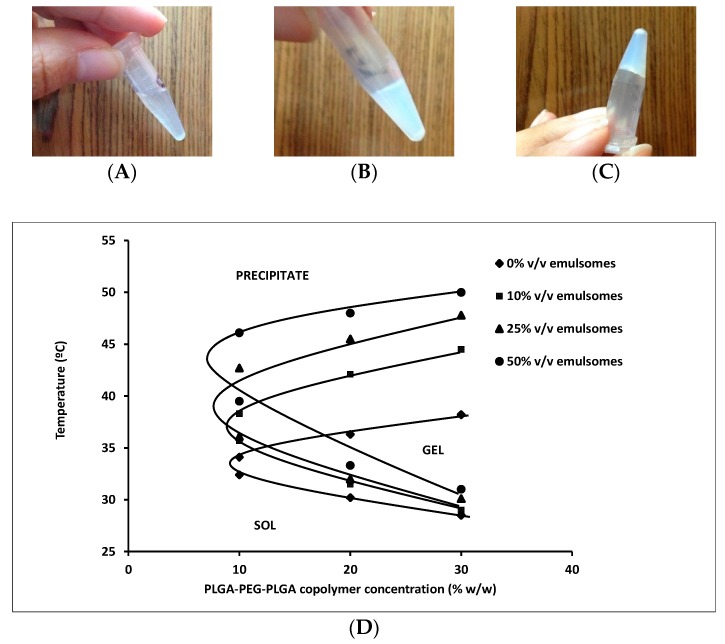
(**A**,**B**) are the photographs of a PLGA-PEG-PLGA copolymer solution prior to sol-gel transition (**A**) and formation of opaque solution (**B**), thermogels produced after sol-gel transition retain their position (**C**). (**D**) is the phase diagrams of polymers prepared from different PLGA-PEG-PLGA copolymer concentrations with different emulsomes loading, showing sol-gel and gel-precipitate transitions).

Before insertion of the copolymer solution in the water bath, and below its transition temperature, the copolymer solution was clear (Figure 2A). Upon heating, the copolymer solutions became opaque without gelation (Figure 2B) or they formed a gel with an absence of flowability upon inversion (Figure 2C). Subsequent temperature decrease transforms the gel into its initial liquid state.

The sol-gel transition temperatures of all the prepared polymer solutions (G1-G16) are given in Table 1. Plain or drug loaded polymer solutions that were prepared from 5 and 10% *w/w* PLGA-PEG-PLGA copolymer (formulations G1, G5, G9, and G13) and (formulations G2, G6, G10, and G14), respectively, remained either clear or they formed turbid solutions showing no gelation till 52 °C. Non-gelling opaque solutions were formed. However, formulations with higher concentrations of PLGA-PEG-PLGA (20 and 30% *w/w*) gelled, completely, at temperatures ranging between 28.50 and 33.37 °C. With temperature rise, up to 52 °C, copolymers prepared from 10, 20, and 30% *w/w* PLGA-PEG-PLGA underwent gel-precipitate transition in which the polymers reverted to a sol state (resol) but as a turbid suspension in which the polymer eventually precipitated. This thermo-reversible behavior of PLGA-PEG-PLGA copolymers has been reported in the literature [23,24,50,51]. Similar to our results, Duvvuri et al., (2005) reported the sol-gel transition temperature of 20–25% *w/v* aqueous polymer solution to be 32 °C [34]. The phase diagram of the copolymer with different emulsome loads is shown in Figure 2D.

The effect of varying PLGA-PEG-PLGA and emulsomes concentrations on the thermogelation temperatures of the copolymer solutions is shown in Table 1. At the same polymer concentration, increasing the emulsomes concentration resulted in a statistically significant increase in the transition temperatures from 30.2 ± 0.05 to 33.4 ± 0.05 °C at 20% *w*/*w* polymer and from 28.5 ± 0.1 to 31 ± 0.0 °C at 30% *w/w* polymer (*p* > 0.05). The increase in emulsomes concentration hinders PLGA interaction leading to retardation in gelation. The hydrophilic Tween 80 coated emulsomes with their polyoxyethylene chains are expected to render the interaction of hydrophobic blocks of PLGA-PEG-PLGA more difficult and they increase transition temperature of the composite. Similar behaviour was recorded upon increasing the concentration of hydrophilic nanoparticles incorporated in PDEGMMA block copolymer based hydrogels [51]. On the other hand, it was also found that increasing the copolymer concentration resulted in a reduction in the phase transition temperatures. On a similar basis, Qiao et al. [45] reported a decrease in gelation temperature that was associated with increasing the same copolymer concentrations from 15 to 25% *w/w*.

Hydrogelation demands two contradictory forces: strong inter-chain interaction in order to form junction points in the hydrogel network, yet the chain should not exclude the solvent (water) to prevent polymer precipitation. The polymer dissolution of the block copolymer at lower temperatures is due to the hydrogen bond between the hydrophilic PEG and water. The rise in temperature weakens the hydrogen bonding prevailing the hydrophobic forces of PLGA blocks, leading to the gel transition state. Accordingly, higher copolymer concentrations enhances the hydrophobic interactions and with the insufficiency of polymer hydration with the available amount of water causes a decrease in the sol-gel transition temperature [52].

In terms of properties of associated copolymer, PLGA-PEG-PLGA triblock copolymers form micellar structures, with a PLGA core and a PEG corona. At a temperature lower than CGT, individual and grouped micelles coexist in the sol state. With temperature rise, the fraction of individual micelles decreases and the size of grouped micelle grows rapidly causing initial sol-gel transition. The aggregation and packing interaction between micelles increase forming an opaque gel (Figure 2). If the temperature is further raised, then the hydrophobic PLGA chains of the micellar core shrink tightly and the hydrophilic PEG blocks undergo dehydration. Highly shrunk micellar groups will eventually precipitate in water, and the system becomes separated into two phases, water and polymer (Figure 1) [24].

#### 3.2.2. Viscosity Measurement

**Figure 3:**
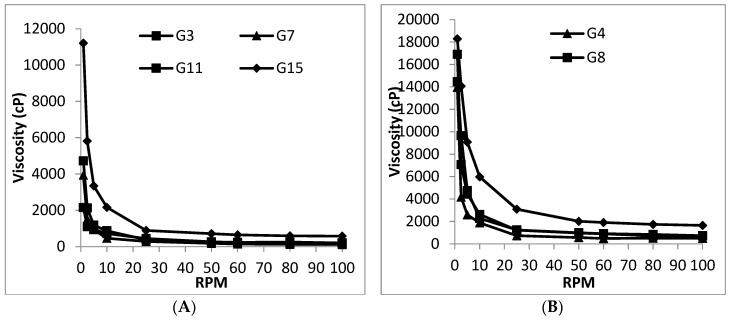
Rheograms of thermogels G3, G7, G11 and G15 prepared from 20% *w/w* PLGA-PEG-PLGA copolymer solution (**A**) and thermogels G4, G8, G12 and G16 prepared from 30% *w/w* PLGA-PEG-PLGA copolymer solution (**B**) in combination with 0, 10, 25, and 50% *v/v* emulsomes measured at 37 °C.

The viscosities of all polymer solutions, post gelation at 37 °C, prepared using 20 and 30% *w/w* PLGA-PEG-PLGA copolymer and 0, 10, 25, and 50% *v/v* emulsomes, and measured at rpms ranging from 1 to 100 are shown in Figure 3. The figure shows that the rheological behavior of both plain and emulsome-loaded thermogels was concentration-dependent. Generally, an increase in viscosity accompanied the increase in copolymer and emulsome concentrations, especially when measured at the lowest rpm due to the preservation of the gel network structure.

Figure 3A shows that at 20% *w/w* copolymer concentration the viscosities were in the order: G15 > G11 > G7 > G3, at lower rpms (*p* < 0.05), directly correlated to with increasing emulsome concentration. However, G15 showed the absolute highest viscosity at all rpm values due to its 50% emulsomal content (*p* < 0.01). A similar trend was observed for G16 containing 30% *w/w* copolymer Figure 3B.

The incorporation of emulsomes will contribute to the formation of a stronger gel matrix. The presence of emulsomes caused a delay in PLGA interaction and micellar formation. Better polymer hydration occurred with the formation of highly viscous gel. A suggestion confirmed by the increase in gelation temperature by the incorporation of emulsomes. Likewise, Guo et al. [53] reported a concentration dependent rheological behavior of methoxyestradiol solid lipid nanoparticles loaded into a PLGA-PEG-PLGA thermosensitive hydrogel accompanied with viscosity increase.

The study also revealed that significantly higher viscosities were encountered (*p* < 0.01) for 30% *w/w* copolymer (formulations: G4, G8, G12, and G16), at all emulsomal concentrations, than for 20% (formulations: G3, G7, G11, and G15). This was an expected behavior because higher copolymer concentration is translated into a denser thermogel matrix, and hence, higher viscosity [38,54]. Due to its high viscosity, G16 emulsomal thermogel was selected for further studies. The G16 emulsomal thermogel showed viscosity values of 3090 cp and 1656 cp at shear rate of 50 and 200 s^−1^ (sneezing produce a maximum shear rate of only >180 s^−1^ [55]). Knowing that IN formulations may leave the nasal cavity by gravity when their viscosity is less than about 2500 centipoise [56], G16 emulsomal thermogel represents a good candidate for IN delivery of OX.

### 3.3. In Vitro Release Study

Three mechanisms for controlling drug release from PLGA based matrices have previously been described, namely (i) Fickian diffusion through the polymer matrix, (ii) diffusion through water-filled pores (aqueous channels) formed by water penetration into the matrix, and (iii) liberation by erosion of the polymer matrix [57]. The actual drug release from these polymer matrices may be controlled by a combination of these three mechanisms [58]. 

**Figure 4:**
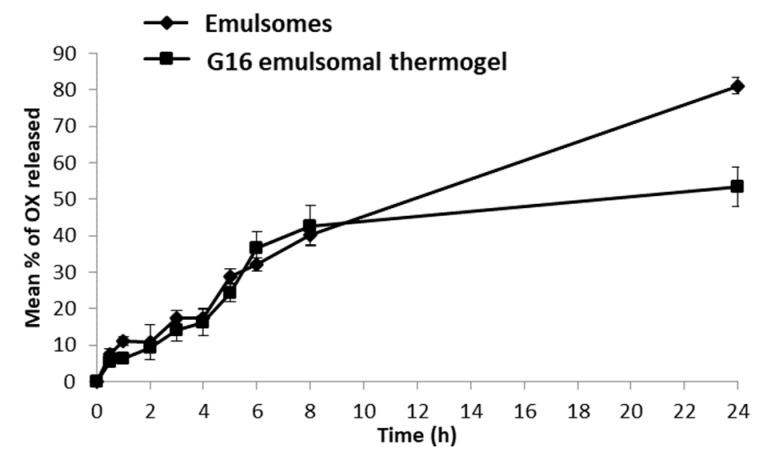
Release profiles of Oxcarbazepine (OX) from emulsomes and G16 emulsomal thermogel.

Figure 4 shows the cumulative average amounts of OX released from emulsomes in comparison with G16 emulsomal thermogel for up to 24 h. Both formulations showed similar release profiles with no significant difference (*p* < 0.05) during the first 8 h. However, after 24 h, the OX release was reduced significantly from 81.1 to 53.5% (*p* < 0.01) in the G16 emulsomal thermogel as compared to the OX-emulsomes. The lack of initial difference in release profile between emulsomes and emulsomal thermogel may be due to the low solubility of OX, which would enhance its retention in both emulsomes and emulsomal thermogel up to 8 h of release. However, after 24 h more OX dissolved from the emulsomal core, and was released directly to the medium in case of emulsomes in comparison with emulsomes dispersed in hydrogel which showed a retarded OX release. Similar behaviour has been shown in situ with emulsomal thermosensitive gel, which was loaded with sparfloxacin for ocular delivery. A release profile that depended on drug solubility for up to 8–10 h was shown, however, when drug release was assessed at longer times it increased dramatically [59]. 

Moreover, drug release from hydrogel depots can be influenced by many factors, including pore size, rate of degradability of the hydrogel, size and morphology of the matrix, concentration of drug, interactions between the hydrogel and the loaded drug delivery system, polymer molecular weight, lactide/glycolide copolymer ratio, as well as matrix fabrication method [60]. 

### 3.4. Mucoadhesion Study

One important design feature of formulations designed for IN administration is the ability to exhibit retention within the nasal cavity with the constant exposure to mucocilliary clearance and enzymatic degradation. Table 2 shows the mucoadhesion parameters (peak detachment, deformation peak, work of adhesion, final load) measured for plain (thermogel G4) and emulsome-loaded PLGA-PEG-PLGA copolymer (G16 emulsomal thermogel), as compared with a 0.5% carbopol gel as a positive standard.

The “work of adhesion”, also termed “adhesiveness”, describes the work that is required to overcome the attractive forces between the surface of the sample and the surface of the probe [61]. This infers that probe removal occurs due to cleavage of both internal bonds within the sample (cohesive bonds), as well as, bonds occurring between the sample and the surface of the probe (adhesive bonds) [62].

The G4 and G16 gels both showed statistically significant higher adhesive properties for all mucoadhesive parameters, when compared to the Carbopol980 gel standard (*p* < 0.05). During optimal contact time between formulation and mucosa that ensures maximal adhesion, gel hydration occurs, which allows for exposure of its adhesive sites. This facilitates interpenetration of the gel adhesive moieties and entanglement with mucus glycoproteins of the nasal mucosa, to a sufficient depth that enables the creation of adhesive bonds [63], thus creating mucoadhesion. On a similar basis, Yu and co-workers proved that PLGA-PEG nanoparticles can penetrate human mucus secretions due to strong interactions with mucin [64]. Besides the results show that the increase in viscosity might help, but it is not crucial for mucoadhesion [65]. Thus, G16 emulsomal thermogel would be expected to show prolonged retention in the nasal cavity through the formation of adhesive bonds with the tissues lining the nasal mucosa. Since the half-life of clearance from the nasal cavity in humans, even for bioadhesive materials is in the order of 1–2 h [66], the differences in OX release after 8 h between the OX-emulsomes and the G16 emulsomal thermogel will likely not affect the nasal absorption of the OX in humans.

It is worthy to note that emulsomes had no influence on the mucoadhesive characters of PLGA-PEG-PLGA copolymer, probably because the binding with mucin occurs via hydrophilic interaction with the PEG chains of the polymer while emulsomes only interact with hydrophobic PLGA domains.

### 3.5. Pharmacokinetic Study 

#### 3.5.1. Plasma Pharmacokinetic Parameters

**Figure 5:**
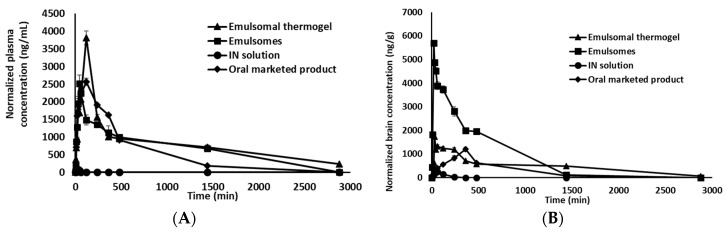
Mean OX concentrations in plasma (**A**) in brain (**B**) of rat groups receiving IN administration of emulsomes, G16 emulsomal thermogel, OX solution and Oral Trileptal^®^ suspension. Data of IN administration of emulsomes, and Oral Trileptal^®^ suspension reproduced with permission from El-Zaafarany et al. [40], Elsevier, 2016.

Nose-to-brain drug delivery represents a potential route for targeting drugs to the CNS and avoiding large systemic losses after drug administration. Figure 5A shows plasma concentration-time profiles of OX after the administration of Trileptal^®^ suspension, IN solution, IN emulsomes, and IN G16 emulsomal thermogel loaded with the drug, with the corresponding PK parameters given in Table 3. Data of OX-emulsomes and Trileptal^®^ suspension were incorporated into the paper for comparative purposes.

In terms of plasma concentrations, the results revealed that the highest *C*_max_ of 3818.8 ng/mL (*p* < 0.01) was encountered after the instillation of IN G16 emulsomal thermogel at a *T*_max_ of 120 min, followed by Trileptal^®^ suspension and IN emulsomes, which reached *C*_max_ value of 2567.6 and 2514.4 ng/mL at *T*_max_ value of 45 and 120 min, respectively, and finally the IN OX solution had the lowest *C*_max_ of 80.9 ng/mL after 20 min. The largest AUC _0–2880min_ was obtained after administration of the IN G16 thermogel, followed by the IN OX emulsomes, Trileptal^®^ suspension then IN OX solution (*p* < 0.05). 

**Figure 6:**
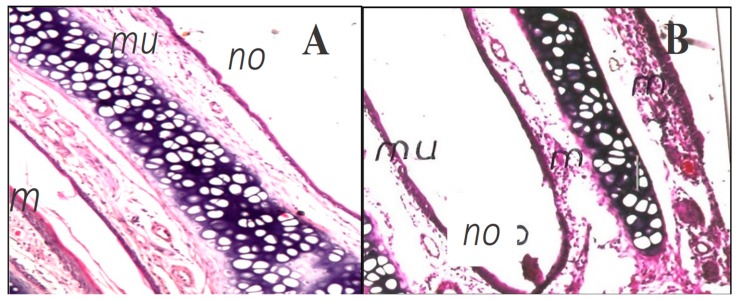
Light micrograph of (**A**) untreated nasal rat epithelium (with saline only) showing normal structure of the nose (no) with intact mucosal epithelium (mu). (**B**) nasal rat epithelium treated with OX-loaded G16 emulsomal thermogel showing intact mucosal epithelium (mu) with minimal focal inflammatory cells infiltration (m). (Maginifcation 16×).

Furthermore, emulsomes and G16 thermogel formulations showed an MRT in plasma 58.7 and 76.5 times that of the IN OX solution respectively. Significantly larger residual amounts of OX after 2880 min were found in the plasma for G16 thermogel than for the OX emulsomes and IN OX solution (*p* < 0.01) (Figure 6). On the other hand, the OX administered IN in emulsomes and G16 thermogel resided in the plasma for up to 34 and 44 h, respectively. The plasma half-life of the G16 thermogel was found to be 1.2 times that of the IN OX emulsomes and significantly higher i.e., 6.4 and 5.3 times that of the IN OX solution and Trileptal^®^ suspension, respectively (*p* < 0.05). 

The long residence in plasma of OX when administered in combination with emulsomes (as opposed to the OX solution) might be explained by the lipophilic nature of the emulsomes that may allow for the partitioning of the OX in the emulsome particles (and/or the free OX) into the nasal epithelial cell membranes and further into the systemic circulation. The high absorption of OX, as shown by the AUC _0–2880min_ and long mean residence time from the IN G16 emulsomal thermogel compared to IN OX-solution could be partly attributed to the high viscosity of the gel formulation which prolonged the contact time with nasal mucosa, thus enhancing the penetration of OX-emulsomes and/or free OX through the nasal mucosa into the systemic circulation [67]. The pharmacokinetic results suggest that the encapsulation of OX in emulsomes slowed its elimination, retained a high concentration in the bloodstream for a longer time period, and increased the circulation time of OX in rats, when compared to the free drug [68].

#### 3.5.2. Brain Pharmacokinetic Parameters

OX concentrations in the brains of rats after the administration of IN OX-emulsomes, IN G16 OX-emulsomal thermogel, IN OX solution, and Trileptal^®^ suspension of OX at predetermined time intervals are depicted in Figure 5B, with the corresponding pharmacokinetic parameters given in Table 3.

The OX concentration in the brain after administration in an IN OX solution was detected after 10 min of administration, showed a low *C*_max_ and reached baseline in 6 h. This observation and the low *C*_max_ in the plasma support the reported low ability of OX to cross mucosal membranes [69,70,71].

OX released from emulsomes and G16 thermogel was detected in the brain only 5 min after administration, but with significantly (*p* < 0.01) higher brain concentration (5 times) for the OX- emulsome formulation, as compared with that of the G16 OX-emulsomal thermogel formulation. Furthermore, the nasal administration of OX-emulsomes resulted in the highest OX brain *C*_max_ which was nearly 3.3, 22.9, and 4.8 times that of G16 OX-emulsomal thermogel, IN solution and Trileptal^®^ suspension, respectively, with earlier *T*_max_ for emulsomes than all tested formulations. In contrast to the plasma data, the OX-emulsomes showed higher brain AUC _0–2880min_ than G16 OX-emulsomal thermogel, IN OX solution and Trileptal^®^ suspension. This could be attributed to the structure of emulsomes that comprises its lipidic phospholipid sheath, the Tween 80 coat and the small size (100–200 nm), all which are contributing factors to enable transcellular diffusion of OX (free or encapsulated) through the blood brain barrier [72]. Detection of OX in the brain within a time frame of 10–20 min after IN administration of emulsomes indicates that direct nose-to-brain transport was a main route for brain uptake from the nasal mucosa [73], in addition to its systemic absorption.

On the other hand, G16 OX-emulsomal thermogel had longer mean residence times in the brain, which exceeded that of the OX-emulsomes, IN OX solution and Trileptal^®^ suspension. Also, the G16 emulsomal thermogel was the only tested formulation in which OX was detected in the brain after 48 h of its IN instillation (data not shown) which could result in a better control of seizures in patients for a longer time period. These results are in accordance with previously reported studies in animals that proved that formulations with a prolonged residence in the nasal cavity using either mucoadhesive systems or gels with very high viscosity can enhance the nose to brain delivery of drugs due to reduced mucociliary clearance and increased residence which ensure higher absorption. Furthermore, it has also been reported that some mucoadhesive polymer-containing systems may directly change epithelial tight junctions and thus, increase drug absorption [74,75].

Although the IN adminstration of emulsomal thermogel showed a lower *C*_max_ in the brain, it showed higher MRT values. It was previously reported that it is more advisable to use extended-action formulations for antiepileptic drugs with short half-lives (OX *t*_1/2_ = 1–5 h) for a more efficient way of treating epilepsy [76].

When compared with formulations that rapidly release antiepileptic drugs, products with extended residence in the brain not only allow for a long dosing interval, which improve patient compliance but also decrease the risk of seizure breakthrough after missing a dose. Furthermore it was found that the use of sustained OX formulations for the treatment of focal epilepsy showed better tolerability and higher maintenance dosages to be reached than immediate action formulations [77].

### 3.6. Histopathological Examination

Nasal histopathology studies on rats were done to check the probable changes in the nasal mucosa caused by G16 emulsomal thermogel, in comparison to normal saline treated nasal epithelium. The control group showed normal, well defined histological structures without any signs of vascular or inflammatory changes (Figure 6A). The histopathology of the treated group revealed some signs of very low toxicity after administration of the G16 OX-emulsome thermogel (Figure 6B). This toxicity was lower than that reported for the drug alone and it is seen here as mild vascular congestion and moderate inflammatory changes around congested vessels.

The safety of PLGA-PEG-PLGA thermogels for a variety of biological applications has been previously reported [32,78,79,80]. Hence, this mild inflammation probably pertains to the effect of the drug on the nasal mucosa due to its controlled release throughout the study, since OX has been reported to have an irritant effect on cells by NADPH dependant DNA intercalation [81]. 

This observation of mild cell toxicity induced by OX is in agreement with previous finding [40,82] that showed OX as an agent that can cause anatomic and organizational disruption in cell layers, nuclear shrinkage, and cell atrophy. Conclusively, these findings would add to our previous finding that emulsomes can shield OX cell damaging effects [40] the ability of G16 emulsomal thermogel composite to further protect nasal cells from the severe effect of OX. Thus, adopted PLGA-PEG-PLGA thermogel matrices could be used to enhance safety and reduce the nasal damage of many other cell membrane damaging drugs.

## 4. Conclusions

A successful PEG-initiated ring opening polymerization of lactide and glycolide to prepare PLGA-PEG-PLGA triblock copolymer was confirmed by NMR, GPC, DSC, and DLS. The copolymer was mixed with emulsomes to form thermoresponsive gels for the nasal delivery of OX. All of the prepared thermoresponsive gels exhibited a reversible thermogelling behavior with visible precipitation of the copolymer at elevated temperatures. Both copolymer and emulsome concentrations contributed, to the formation of thermogels with high viscosities. Sequestration of emulsomes in 30% thermogel resulted in retardation of OX release, increased gel matrix viscosity, but did not affect mucoadhesive properties as compared to thermogel alone. The OX-emulsome thermogel dramatically increased the systemic OX absorption but did not improve the OX brain concentration in terms of *C*_max_ and AUC as compared to the OX-emulsomes alone. However, the emulsomal thermogel, extended the presence of OX in the brain to up to 48 h, which could offer a better control of seizures for a longer time period. A fact that needs to be investigated further.

## Figures and Tables

**Table 1:** Coding, compositions and gelation temperatures of prepared thermogels.

Emulsome Concentration % *v/v*	Gelation Temperature (°C ± SD)			
	PLGA-PEG-PLGA Copolymer (% *w*/*w*)			
	5%	10%	20%	30%
0%	G1 No gelation	G2 Opaque solution	G3 30.2 ± 0.05 °C	G4 28.5 ± 0.1 °C
10%	G5 No gelation	G6 Opaque solution	G7 31.5 ± 0.1 °C *	G8 29.0 ± 0.06 °C *
25%	G9 No gelation	G10 Opaque solution	G11 32.0 ± 0.06 °C *	G12 30.1 ± 0.05 °C *
50%	G13 No gelation	G14 Opaque solution	G15 33.4 ± 0.05 °C *	G16 31.0 ± 0.0 °C *

* Significantly different (*p* < 0.05) from emulsome free gel.

**Table 2:** Mucoadhesion parameters measured for plain (G4) and G16 emulsome-loaded PLGA-PEG-PLGA thermogel compared with a standard Carbopol 980 gel.

Parameter	Plain G4 Thermogel (Mean ± SD)	G16 Emulsomal Thermogel (Mean ± SD)	Carbopol 980 Gel (Mean ± SD)
Peak detachment force (N)	21.9 ± 1.7 *	18.7 ± 2.3 *	10.52 ± 0.1
Deformation peak (mm)	10.5 ± 2 *	8.2 ± 1.3	5.4 ± 0.8
Work of adhesion (mJ)	788.3 ± 23.7 *	746.6 ± 52.2 *	304.8 ± 35
Final load (N)	20.2 ± 1.7	17.84 ± 2.9	15.41 ± 1.9

* Significantly different from Carbopol 980 gel (*p* < 0.05).

**Table 3:** Pharmacokinetic parameters for OX in rat plasma/brain following the IN administration of emulsomes, G16 emulsomal thermogel, OX solution and Oral Trileptal^®^ suspension.

Parameter	Plasma				Brain			
	IN Emulsomes *	IN Emulsomal Thermogel	IN OX Solutions	Trileptal^®^ Suspension *	IN Emulsomes *	IN Emulsomal Thermogel	IN OX Solutions	Trileptal^®^ Suspension *
*C*_max_ (ng/mL)/(ng/g)	2514.4	3818.8	80.9	2567.6	5699.9	1733.2	249.1	1198.9
*T*_max_ (min)	45	120	20	120	20	30	60	360
AUC_0–2880min_ (µg/mL·min)/(µg/g·min)	1943.4	2757.7	111.2	1524.6	2445.1	1440.5	57.7	742.8
*T*_1/2_ (min)	1581.8	1919.6	302.1	364	250.9	734	197.4	285.6
MRT (min)	2024.3	2638.1	34.5	465.4	378	1007.8	95.4	504.5

* Data reproduced with permission from El-Zaafarany et al. [40], Elsevier, 2016.

## Supplementary Materials

The following are available online at http://www.mdpi.com/1999-4923/10/4/217/s1, Figure S1. Synthesis of PLGA-PEG-PLGA triblock copolymer by ring opening polymerization of glycolide, d,l-lactide and polyethylene glycol (PEG); Figure S2. ^1^H-NMR spectrum of PLGA-PEG-PLGA tri-block copolymer with (a) and (d) pertaining to lactide methyl and methane protons, (b) pertaining to ethylene oxide methene protons and (c) pertaining to glycolide methene protons; Figure S3. GPC chromatograms of PEG and PLGA-PEG-PLGA copolymer; Figure S4. DSC thermograms of PEG polymer and the synthesized PLGA-PEG-PLGA tri-block copolymer; Figure S5. Size distribution of PLGA-PEG-PLGA micellar solution (20% *w*/*v* in deionized water) measured at temperatures in the range of 10 to 45 °C; Figure S6. (A)Temperature sweep data that shows variation in viscosity and phase angle with temperature for PLGA-PEG-PLGA copolymer samples prepared in concentrations of 15, 20, and 25% *w*/*v* in deionized water (B) Time sweep data that shows variation in elastic modulus, viscous modulus and viscosity of PLGA-PEG-PLGA copolymer sample with time (C) Frequency sweep data that shows variation in elastic and viscous modulus with frequency for PLGA-PEG-PLGA copolymer samples prepared in concentrations of 15, 20, and 25% *w*/*v* in deionized water.
