# Supplementary material for: A Tailored Thermosensitive PLGA-PEG-PLGA/Emulsomes Composite for Enhanced Oxcarbazepine Brain Delivery via the Nasal Route
Source: Pharmaceutics. 2018 Nov 5;10(4):217. doi: 10.3390/pharmaceutics10040217 (PMC6321319; doi:10.3390/pharmaceutics10040217)
Supplement: Supplementary file 1 [file pharmaceutics-10-00217-s001.pdf]

pharmaceutics

MDPI

Supplementary Materials: A tailored Thermosensitive PLGA-PEG-PLGA/Emulsomes Composite for Enhanced Oxcarbazepine Brain Delivery via Nasal Route

Ghada M. El-Zaafaran, Mahmoud E. Soliman, Samar Mansour, Marco Cespi, Giovanni Filippo Palmieri[2], Lisbeth Illum, Luca Casettari and Gehanne A.S. Awad

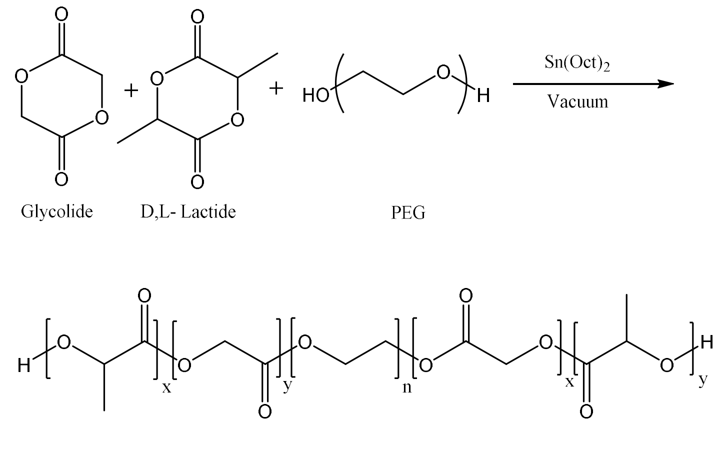

Figure S1. Synthesis of PLGA-PEG-PLGA triblock copolymer by ring opening polymerization of glycolide, D,L-lactide and polyethylene glycol (PEG).

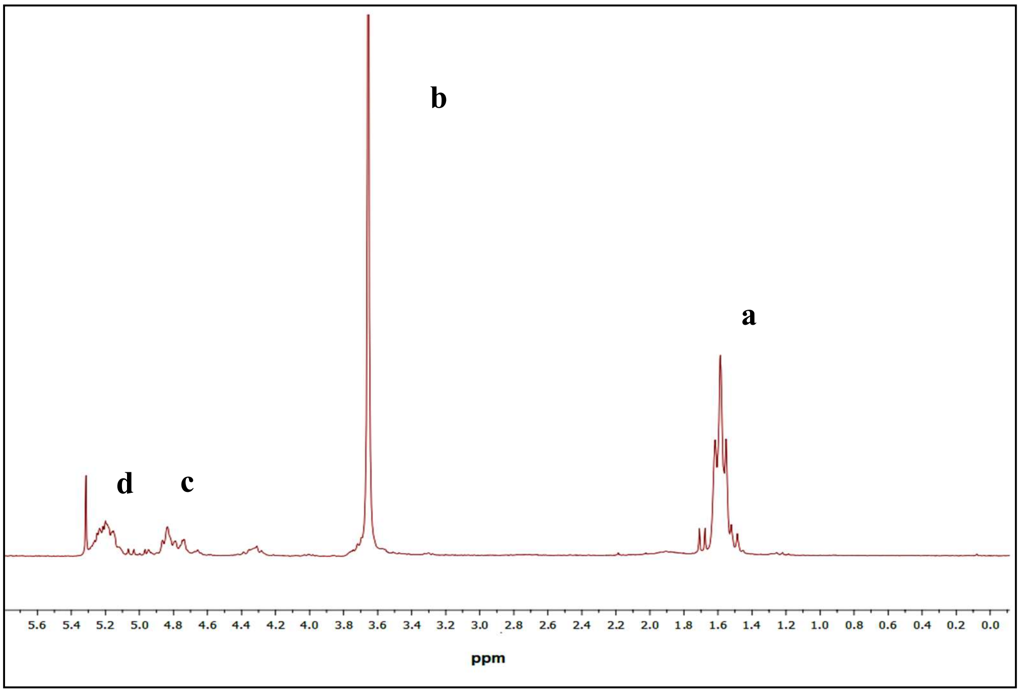

Figure S2. ^{1}H-NMR spectrum of PLGA-PEG-PLGA tri-block copolymer with (**a**) and (**d**) pertaining to lactide methyl and methane protons, (**b**) pertaining to ethylene oxide methene protons and (**c**) pertaining to glycolide methene protons.

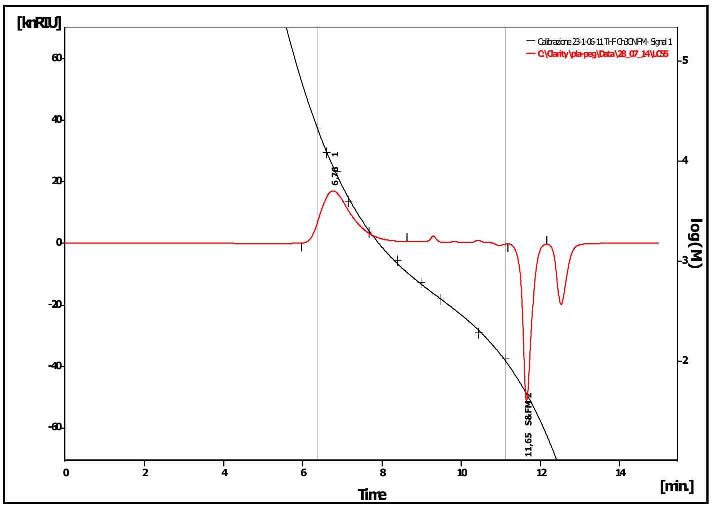

Figure S3. GPC chromatograms of PEG and PLGA-PEG-PLGA copolymer.

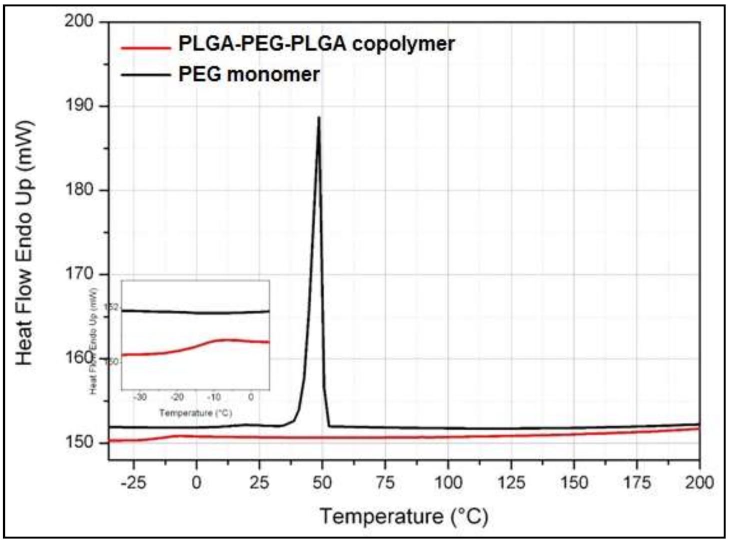

Figure S4. DSC thermograms of PEG polymer and the synthesized PLGA-PEG-PLGA tri-block copolymer.

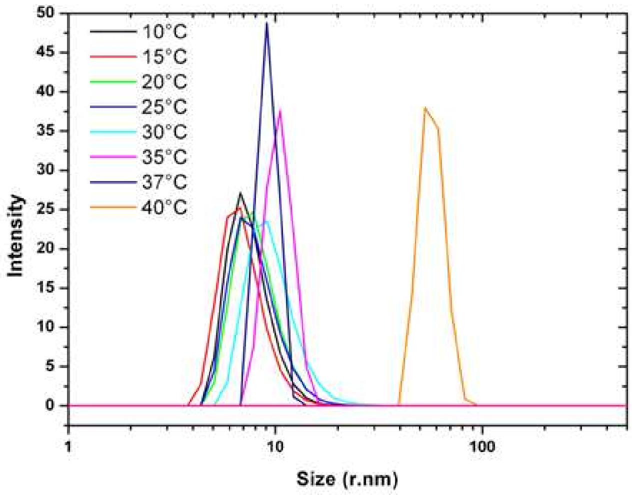

Figure S5. Size distribution of PLGA-PEG-PLGA micellar solution (20 % *w*/*v* in deionized water) measured at temperatures in the range of 10 to 45 °C

Rheological Characterization

The rheological analysis of a PLGA-PEG-PLGA triblock copolymer sample (20 % *w/v* in deionized water) was performed using a rheometer with geometry flat cone (4/40) and gap 150 μm operating in oscillatory mode. The analyzed rheological parameters included; elastic modulus (G'), viscous modulus (G''), the phase angle (tan δ) and complex viscosity (Pa.sec). The storage modulus G' represents the elastic behaviour of the sample and the loss modulus G'' denotes the viscous behaviour of the sample. The phase angle is calculated from G' and G'' as follows:

$$an\ \delta = \frac{G''}{G'}$$

The rheological tests performed were:

Temperature Sweep

A temperature sweep test was performed on polymer samples with PLGA-PEG-PLGA concentrations of 15, 20 and 25 % *w/v* in deionized water, in order to monitor the variation in G', G'' and tan δ with temperature. The temperature was varied from 5 to 55 °C in increments of 1 °C/min at a constant frequency of 1 Hz and stress of 5 Pa.

Time Sweep

A time sweep test was conducted for 30 min while maintaining a constant frequency of 1 Hz, stress of 5 Pa and temperature at 37 °C. This test determines the variation in the above-mentioned rheological parameters by prolonging the analysis for a certain period of time in order to check the rheological stability of the sample or identify the time required to achieve this stability.

Frequency Sweep

A frequency sweep test was done to analyse the effect of oscillation frequency on the rheological parameters of the system. This test was performed by varying the frequency from 0.01 to 10 Hz while maintaining constant stress of 5 Pa and the temperature at 37 °C, on PLGA-PEG-PLGA samples with concentrations of 15, 20 and 25 % *w/v* in deionized water.

(A)

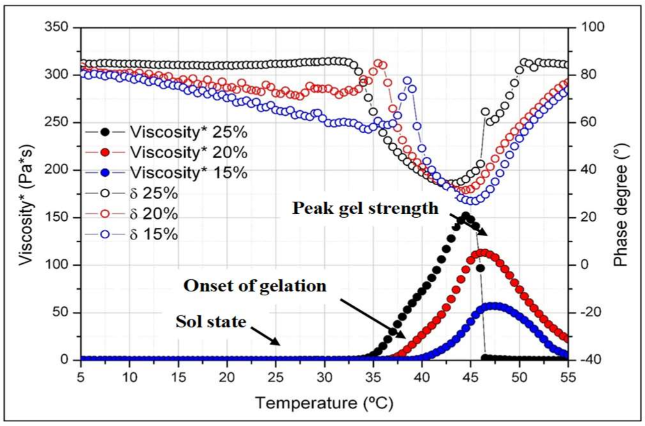

Figure S6. *Cont.*

(B)

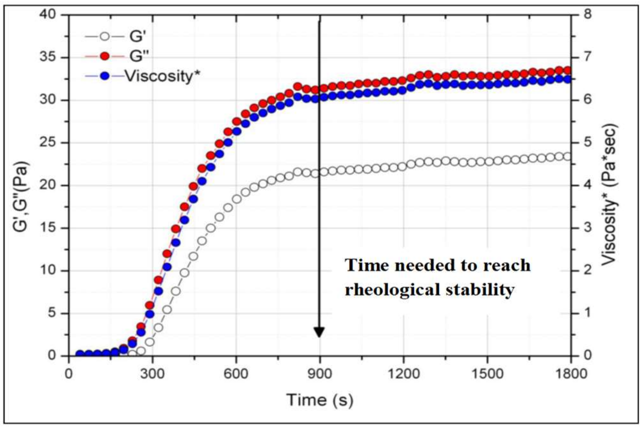

(C)

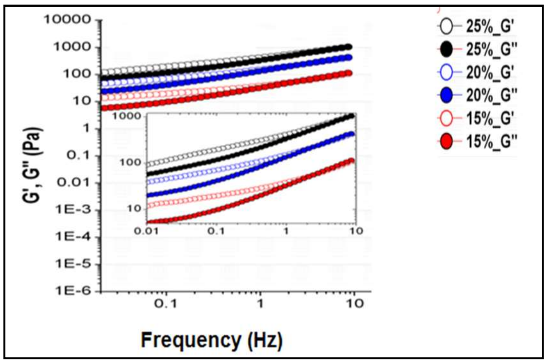

Figure S6: (**A**)Temperature sweep data that shows variation in viscosity and phase angle with temperature for PLGA-PEG-PLGA copolymer samples prepared in concentrations of 15, 20 and 25 % w/v in deionized water (**B**) Time sweep data that shows variation in elastic modulus, viscous modulus and viscosity of PLGA-PEG-PLGA copolymer sample with time (**C**) Frequency sweep data that shows variation in elastic and viscous modulus with frequency for PLGA-PEG-PLGA copolymer samples prepared in concentrations of 15, 20 and 25 % *w/v* in deionized water
